# Hypothalamic subregional volume in relation to VSS-C total symptom burden in women with migraine: an exploratory cross-sectional MRI study

**DOI:** 10.3389/fneur.2026.1931533

**Published:** 2026-09-21

**Authors:** Linyi Che, Wanqun Hu, Xiaoping Fan, Qinlian Huang, Ruonan Zhang, Yuhui Xu, Liang Dong, Li Jiang, Jiying Zhou

**Affiliations:** 1 Department of Neurology, The First Affiliated Hospital of Chongqing Medical University, Chongqing, China; 2 Department of Neurology, Chongqing Jiulongpo District Science City People's Hospital, Chongqing, China; 3 Phase I Clinical Research Center, The First Affiliated Hospital of Chongqing Medical University, Chongqing, China; 4 Department of Rehabilitation Medicine, Key Laboratory of Physical Medicine and Precision Rehabilitation of Chongqing Municipal Health Commission, The First Affiliated Hospital of Chongqing Medical University, Chongqing, China

**Keywords:** dizziness, hypothalamus, migraine, structural MRI, Vertigo Symptom Scale, vestibular symptoms

## Abstract

**Background:**

Dizziness, vertigo, and related autonomic symptoms are common in migraine, but whether their combined symptom-frequency burden is associated with hypothalamic subregional structure remains unclear.

**Methods:**

This exploratory cross-sectional secondary analysis included 39 women with ICHD-3-defined migraine. All source T1-weighted images were processed uniformly with FreeSurfer 7.4.1 and automated hypothalamic subunit segmentation. Blinded quality control was performed without clinical data and independently confirmed by a neuroradiologist. The exposure was VSS-C total score, a composite of vertigo-balance and autonomic-anxiety symptom frequency. Models for five bilateral subunits and whole-hypothalamus volume adjusted for age, estimated total intracranial volume, and baseline monthly migraine days; Benjamini-Hochberg false-discovery-rate correction was applied across the six outcomes. Outcome-specific quality-control denominators were 37 or 39.

**Results:**

Anterior-superior hypothalamic volume was associated with VSS-C total score (n = 39; β = 0.165 mm^3^ per point, 95% confidence interval 0.064 to 0.266, *p* = 0.0021; BH-FDR-adjusted *p* = 0.0127). The association remained after restricting all six outcomes to the same 37 participants (BH-FDR-adjusted *p* = 0.0334). Across the observed 20-point VSS-C interquartile range, the coefficient corresponded to 3.31 mm^3^, or 8.54% of mean anterior-superior volume. DHI identified the same subregion in a separately corrected supporting analysis, but mutual adjustment attenuated both coefficients.

**Conclusion:**

Higher VSS-C total symptom burden was associated with larger anterior-superior hypothalamic volume in women with migraine. This result identifies a subregional target for prospective replication with component-level symptom assessment and formal vestibular phenotyping.

## Background

Migraine is defined clinically by recurrent headache attacks and associated sensory or gastrointestinal symptoms, but many patients also report dizziness, vertigo, motion sensitivity, or broader audiovestibular complaints (1–5). Formal vestibular migraine criteria are intentionally specific (2). In contrast, clinical migraine cohorts can be characterized dimensionally without assuming that every participant meets the categorical diagnosis. For structural imaging, such a measure can test associations across symptom frequency without implying formal vestibular-migraine adjudication.

The hypothalamus is a plausible structure for such a question. Migraine imaging and experimental literature have linked hypothalamic function and structure to homeostatic regulation, autonomic control, attack dynamics, and interoceptive state regulation (6–14). Vestibular symptoms also interact with autonomic and anxiety-related balance systems (15–18). These links do not identify a specific hypothalamic nucleus, but they support asking whether imaging-defined hypothalamic subregional measures vary with Chinese Vertigo Symptom Scale (VSS-C) total symptom burden within migraine.

Existing structural MRI studies have characterized hypothalamic differences using case–control structural covariance and microstructural approaches (8–10). These designs leave a narrower question about symptom dimensionality within migraine: whether a recorded composite symptom-frequency score varies across automated hypothalamic subregions. VSS-C total score addresses that question when subscale-level data are unavailable, but it cannot isolate the vertigo-balance contribution from its autonomic-anxiety component or serve as a vestibular-migraine diagnosis.

We therefore examined whether VSS-C total symptom burden (19, 20) was associated with FreeSurfer-defined hypothalamic subregional volume in women with migraine. The Dizziness Handicap Inventory (DHI) was analyzed as a correlated supporting measure of dizziness-related disability (21). Because no directional hypothesis was prespecified for subregional volume and the dataset did not include formal vestibular-migraine adjudication for all participants, the study was framed as an exploratory symptom-dimensional magnetic resonance imaging (MRI) analysis, not as a vestibular migraine diagnostic study.

## Methods

### Study design and participants

This was a retrospective cross-sectional secondary analysis of a prospectively maintained clinical and imaging migraine cohort from the Headache Clinic of the First Affiliated Hospital of Chongqing Medical University. The parent protocol supported clinical and neuroimaging assessment of migraine; the hypothalamic subregional VSS-C hypothesis was developed after cohort data collection. Migraine was diagnosed by headache specialists according to International Classification of Headache Disorders, third edition (ICHD-3), criteria (1).

The secondary-analysis dataset comprised 42 participants with clinical and MRI data (41 women and 1 man). Because the available sample included only one man, sex-adjusted or sex-stratified inference was not estimable. The analytic cohort was limited to women before image review and model fitting; no inference is made about men or sex differences. All 41 women underwent blinded image and segmentation review. Two scans showed gross segmentation or anatomical-alignment failure and were excluded, leaving 39 women. The parent-cohort recruitment denominator and earlier selection pathway were unavailable. Supplementary Figure S1 therefore begins with the 42 participants in the recoverable dataset. No formal prospective sample-size calculation was performed.

The parent study and the present secondary analysis were approved by the Ethics Committee of the First Affiliated Hospital of Chongqing Medical University (approval Nos. CY2020-594 and 2026–0357-01), and all participants provided written informed consent at enrollment. The report was prepared with reference to STROBE and SAGER recommendations (22, 23).

### Clinical variables

Questionnaire-based variables were collected on the day of MRI acquisition. The administered Vertigo Symptom Scale form matched the published Chinese version (VSS-C) (19, 20). It contains 22 questions comprising 34 scored items. Each item is rated from 0 (never) to 4 (very often) according to symptom frequency during the preceding 12 months, or since symptom onset when symptoms had been present for less than 1 year, giving a theoretical total range of 0 to 136. The scale contains a 19-item vertigo-balance component and a 15-item autonomic-anxiety component. The recorded total therefore represents composite symptom-frequency burden rather than a pure vestibular measure. Item-level and subscale responses were unavailable for the full cohort, so their contributions could not be separated. VSS-C total scores were available for all 39 participants and were not imputed. DHI total score was analyzed as a related measure of dizziness-associated disability (21).

Additional clinical variables included age, body mass index (BMI), headache disease duration, baseline monthly migraine days (MMD), baseline monthly headache days, Hospital Anxiety and Depression Scale (HADS), Migraine Disability Assessment (MIDAS), six-item Headache Impact Test (HIT-6), Pittsburgh Sleep Quality Index (PSQI), menstrual-related headache status, and preventive-treatment history. Baseline MMD was diary-derived and entered as a main-model covariate because migraine frequency could confound the relation between VSS-C total score and volume. Diary-quality thresholds and exact treatment exposure during the diary interval were unavailable for analysis.

The parent protocol described clinical exclusion of known vestibular disorders, but the analysis dataset did not contain a standardized vestibular test battery or participant-level diagnostic adjudication. Formal vestibular-migraine adjudication and several additional migraine, treatment, attack-state, and reproductive-hormone fields were unavailable or insufficiently harmonized for modeling (Supplementary Table S4).

### MRI acquisition and hypothalamic volumetry

MRI data were acquired on a Philips Ingenia DNA 3.0-T scanner with a 32-channel head coil. The structural sequence was a 3D T1-weighted turbo spin-echo acquisition with repetition time/echo time = 600/28 ms, excitation flip angle = 90 degrees, field of view = 280 × 280 mm, acquisition matrix = 280 × 280, 327 reconstructed sections, and reconstructed voxel dimensions of 1.0 × 1.0 × 0.5 mm. The protocol aimed to schedule MRI when participants were headache-free and required avoidance of acute analgesics, sedative or hypnotic medication, caffeine-containing drinks, and alcohol for 24 h before scanning. Exact peri-ictal timing was not harmonized for analysis.

Source T1 images were available for all 39 women and processed uniformly with FreeSurfer version 7.4.1. For each de-identified subject, whole-brain preprocessing was initialized with recon-all -autorecon1; the subject-directory hypothalamic module was then run with mri_segment_hypothalamic_subunits. The module operated on the FreeSurfer bias-corrected mri/nu.mgz and generated official soft-segmentation volume outputs (24, 25). The reconstructed scanner image was not supplied directly to the hypothalamic module. Processing was performed in a fixed container image (SHA-256 digest 10b6468cbd9fcd2db3708f4651d59ad75d4da849a2c5d8bb6dba217f08b8c46b). The official labels were anterior-inferior, anterior-superior, inferior tubular, superior tubular, and posterior (25). Left and right values were summed to obtain bilateral subunit volumes, and whole-hypothalamus volume was retained. eTIV was retained from the original participant-matched analysis export as a fixed covariate and was not recomputed in the current imaging run. All reported hypothalamic outcome volumes came from this uniform processing route. A descriptive comparison with estimates from historical volumes is provided in Supplementary Table S5.

Segmentation quality control was completed before clinical linkage. All 39 de-identified cases were reviewed in randomized order without names, VSS-C, DHI, MMD, diagnosis details, or other clinical variables. Canonical sagittal, coronal, and axial overlays were inspected for image-segmentation geometry mismatch, gross processing failure, anatomical misalignment, anatomically implausible labeling, major hypothalamic-mask boundary displacement, and visible image artifact that compromised segmentation plausibility. Objective image and label checks were used as review prompts rather than automatic exclusion criteria. A neuroradiologist independently reviewed the complete de-identified overlay set without access to clinical variables and agreed with the adjudication. All 39 cases passed whole-subject QC. Two cases lacked the right anterior-inferior label in both the official volume output and hard-label segmentation; their bilateral anterior-inferior outcomes were set to unavailable, while their other subregional outcomes were retained. No label was manually repaired. A common 37-participant analysis and a whole-volume analysis excluding both local exceptions were specified as sensitivities. Figure 1 shows the first case in the randomized processing order, selected before clinical linkage, as representative QC documentation; it is not a formal measurement-reliability analysis.

**Figure 1:**
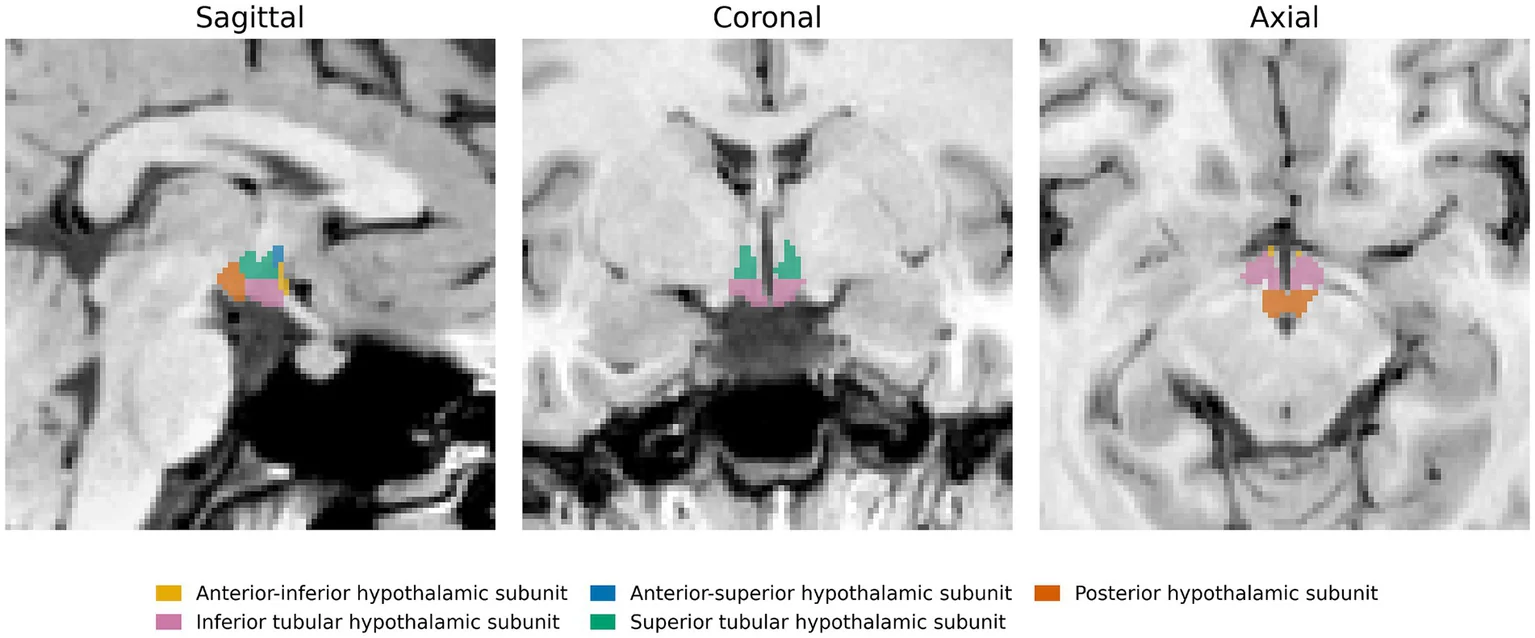
Representative analyzed hypothalamic segmentation used for quality-control documentation. The displayed case was the first participant in the randomized processing order and was selected before clinical linkage. Colored overlays show the five automated subunit labels on the FreeSurfer-processed T1-weighted image in sagittal, coronal, and axial planes. Left and right labels of the same subunit share one color. The image comes from the same uniform FreeSurfer 7.4.1 processing route used to generate the analyzed volumes. It documents the visual QC procedure and is not a formal estimate of segmentation accuracy or inter-rater reliability.

### Statistical analysis

Continuous variables are summarized as mean ± standard deviation or median (interquartile range), as appropriate. The primary exposure was continuous VSS-C total score. Six ordinary least-squares models related VSS-C to bilateral anterior-inferior, anterior-superior, inferior tubular, superior tubular, posterior, and whole-hypothalamus volume, with adjustment for age, eTIV, and baseline MMD. The primary analysis used all QC-valid observations for each outcome (*n* = 37 for anterior-inferior; *n* = 39 for the other outcomes). Benjamini-Hochberg false-discovery-rate (BH-FDR) correction was applied across these six models. A common QC-valid sensitivity repeated the six models in the same 37 participants.

DHI models were supporting analyses and formed a separate six-outcome BH-FDR family. The correlation between VSS-C and DHI was estimated with Spearman’s rho. Mutual-adjustment models for the anterior-superior outcome examined overlap between the two scores; they were interpreted as construct-overlap analyses rather than independent confirmation.

For the anterior-superior outcome, a sequential model first adjusted for age and eTIV and then added baseline MMD on the same 39 participants. Robustness checks comprised HC3 inference across all six VSS-C outcomes, leave-one-out refitting, exclusion of the observation with maximum Cook’s distance, Huber robust regression, residual and heteroscedasticity checks, and variance inflation factors. Same-cohort covariate sensitivities added age squared, HADS, or BMI; the menstrual-related-headache sensitivity used the 38 participants with that field available. These selected-outcome checks assess stability and do not constitute additional multiplicity-controlled confirmation. Two-sided *p* < 0.05 was used for nominal interpretation of supporting analyses; the primary family was interpreted by BH-FDR-adjusted *p* values. Analyses were conducted in Python 3 using pandas, NumPy, SciPy, statsmodels, and Matplotlib.

## Results

### Cohort characteristics and outcome availability

The final cohort comprised 39 women with complete VSS-C, DHI, age, eTIV, and baseline-MMD data (Table 1; Supplementary Figure S1). All 39 source T1 images completed uniform processing and passed whole-subject blinded QC. Two local right anterior-inferior label exceptions reduced the bilateral anterior-inferior denominator to 37; the other five outcomes retained n = 39.

**Table 1:** Final analytic cohort characteristics.

Characteristic	Value
Women, n (%)	39/39 (100.0%)
Age, years	39.1 ± 10.3
BMI, kg/m^2^	21.7 ± 2.7
Disease duration, years	16.0 (12.5–25.0)
Baseline monthly migraine days	13.0 (6.0–20.0)
Baseline monthly headache days	16.0 (8.0–22.0)
Baseline MMD ≥ 15 days, n (%)	19/39 (48.7%)
VSS-C total score	22.0 (12.5–32.5)
VSS-C observed range	0–76
DHI total score	29.0 (11.0–51.0)
DHI observed range	0–86
Car sickness, n (%)	23/39 (59.0%)
HADS total score	13.0 (10.5–15.0)
MIDAS total score	19.0 (11.0–33.0)
HIT-6 score	63.0 (54.5–70.5)
PSQI score	7.0 (5.0–11.0)
Menstrual-related headache, n (%)	23/38 (60.5%)
Preventive-treatment history, n (%)	25/39 (64.1%)
eTIV, mm^3^	1,164,634.1 ± 172,955.5
Anterior-superior volume, mm^3^	38.72 ± 6.60
Anterior-inferior volume, mm^3^	19.90 ± 6.65 (n = 37)
Superior tubular volume, mm^3^	188.71 ± 26.94
Inferior tubular volume, mm^3^	242.29 ± 38.54
Posterior volume, mm^3^	209.45 ± 24.44
Whole hypothalamus volume, mm^3^	698.38 ± 83.65

Values are mean ± standard deviation, median (interquartile range), or n (%). Anterior-inferior volume is based on the 37 participants with both side labels available. BMI, body mass index; DHI, Dizziness Handicap Inventory; eTIV, estimated total intracranial volume; HADS, Hospital Anxiety and Depression Scale; HIT-6, six-item Headache Impact Test; MIDAS, Migraine Disability Assessment; MMD, monthly migraine days; PSQI, Pittsburgh Sleep Quality Index; VSS-C, Chinese version of the Vertigo Symptom Scale.

### Main VSS-C models

Higher VSS-C total score was associated with larger anterior-superior hypothalamic volume (*n* = 39; *β* = 0.165 mm^3^ per point, 95% CI 0.064 to 0.266, *p* = 0.0021; BH-FDR-adjusted *p* = 0.013; Table 2). The other five outcomes were not associated after correction. Restricting all six outcomes to the common 37-participant cohort retained the anterior-superior association (*β* = 0.156, 95% CI 0.049 to 0.262, *p* = 0.0056; BH-FDR-adjusted *p* = 0.033). Across the observed VSS-C interquartile range from 12.5 to 32.5 points, the main coefficient corresponded to 3.31 mm^3^, or 8.54% of mean anterior-superior volume. Figure 2 displays standardized coefficients across the six outcomes, and Figure 3 shows the focal partial-regression relation.

**Table 2:** Primary continuous VSS-C models after outcome-level quality control.

Outcome	n	β (95% CI), mm^3^ per point	*p*	BH-FDR-adjusted P	Partial R^2^
Anterior-superior	39	0.165 (0.064 to 0.266)	0.0021	0.013	0.246
Anterior-inferior	37	0.018 (−0.108 to 0.145)	0.768	0.909	0.003
Superior tubular	39	0.231 (−0.248 to 0.709)	0.334	0.501	0.027
Inferior tubular	39	0.498 (−0.133 to 1.130)	0.118	0.288	0.07
Posterior	39	0.024 (−0.392 to 0.439)	0.909	0.909	0
Whole hypothalamus	39	0.971 (−0.348 to 2.290)	0.144	0.288	0.062

Each model adjusted for age, eTIV, and baseline MMD. β is the adjusted volume difference in mm^3^ per 1-point higher VSS-C total score. BH-FDR correction was applied across all six outcomes. CI, confidence interval; eTIV, estimated total intracranial volume; MMD, monthly migraine days; VSS-C, Chinese version of the Vertigo Symptom Scale.

**Figure 2:**
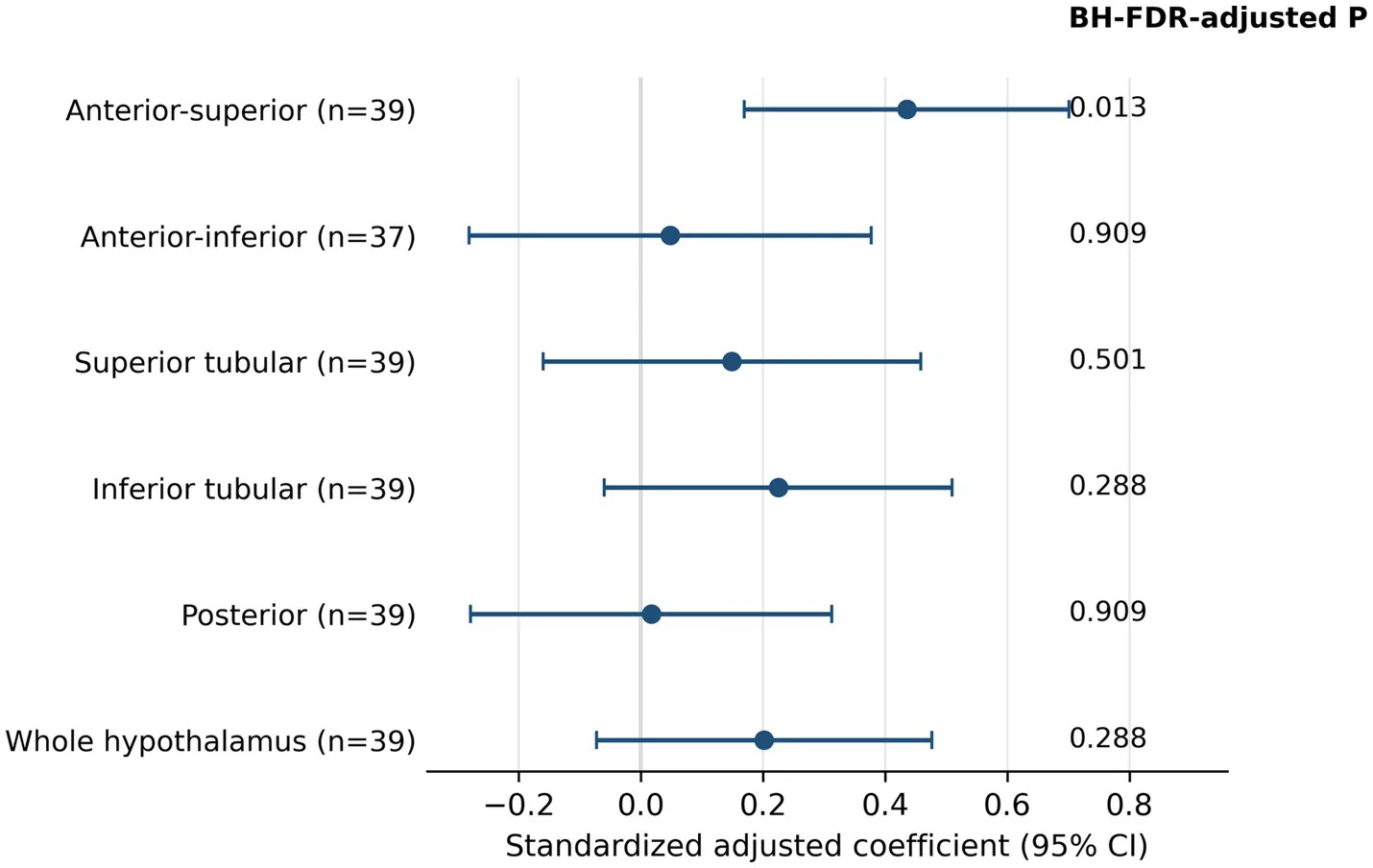
Adjusted associations between VSS-C total score and six hypothalamic volumes. Points and horizontal bars show standardized coefficients and 95% confidence intervals from models adjusted for age, eTIV, and baseline MMD. Outcome-specific QC denominators are shown beside each label. The right-hand column reports BH-FDR-adjusted *p* values across the six conventional ordinary least-squares models. Standardization is used only to compare effect scale across outcomes; unstandardized estimates are reported in Table 2. BH-FDR, Benjamini-Hochberg false-discovery-rate; eTIV, estimated total intracranial volume; MMD, monthly migraine days; VSS-C, Chinese version of the Vertigo Symptom Scale.

**Figure 3:**
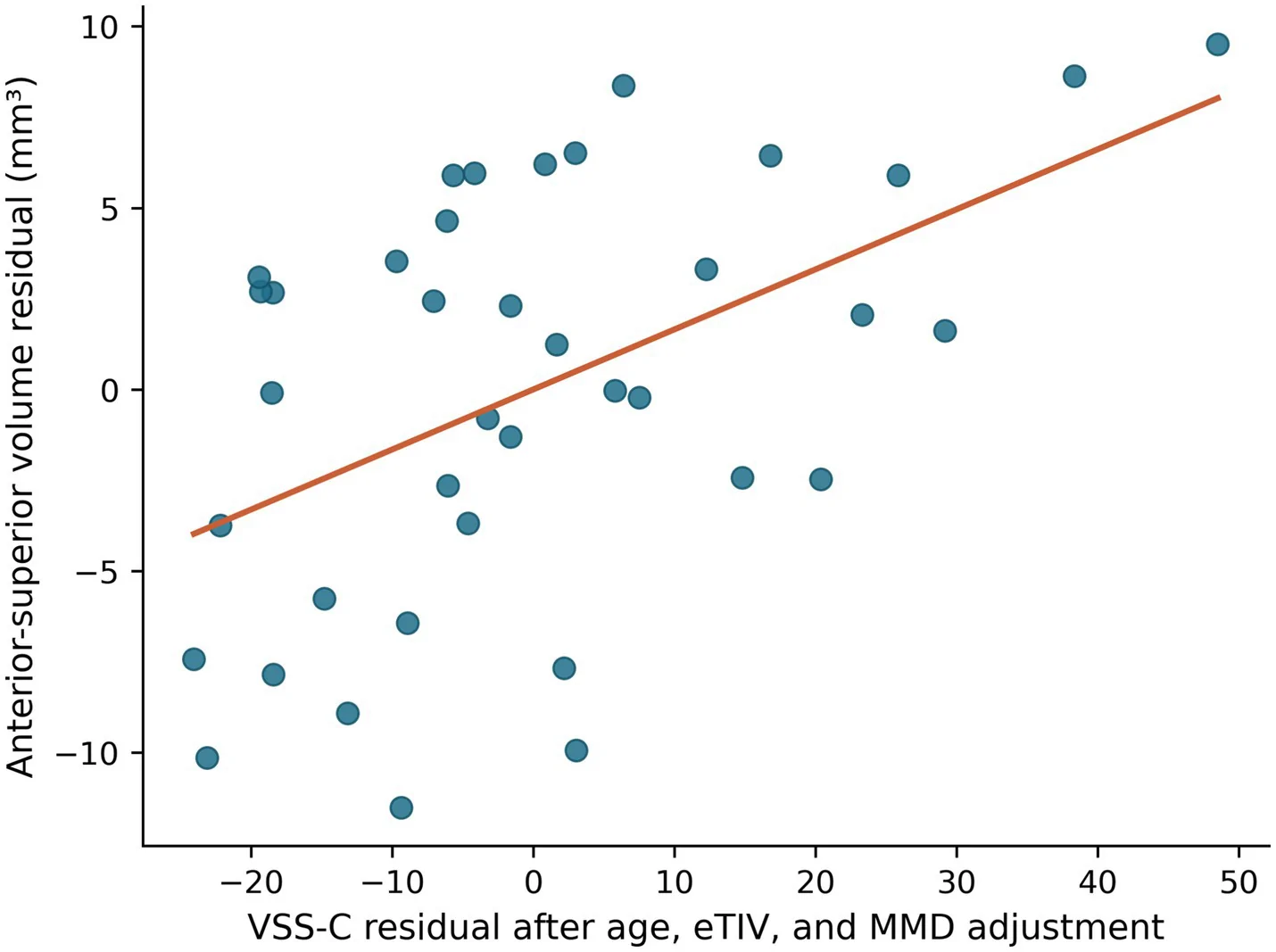
Adjusted association between VSS-C total score and anterior-superior hypothalamic volume (*n* = 39). Both axes show residuals after adjustment for age, eTIV, and baseline MMD. The line is the residual-on-residual fit. This is an adjusted display rather than a plot of raw scores and volumes. Model uncertainty is reported in Tables 2, 3. eTIV, estimated total intracranial volume; MMD, monthly migraine days; VSS-C, Chinese version of the Vertigo Symptom Scale.

### Supporting DHI results and robustness analyses

DHI total score was associated with the same anterior-superior location in its separate supporting family (*n* = 39; *β* = 0.114, 95% CI 0.042 to 0.185, *p* = 0.0027; BH-FDR-adjusted *p* = 0.016; Supplementary Table S2). VSS-C and DHI were correlated (Spearman ρ = 0.661, *p* < 0.001). In the mutual-adjustment model, the VSS-C coefficient was 0.104 (95% CI −0.026 to 0.234, *p* = 0.113) and the DHI coefficient was 0.067 (95% CI −0.025 to 0.158, *p* = 0.147), indicating shared rather than independent signal (Supplementary Table S2).

The age-plus-eTIV coefficient was 0.165 mm^3^ per point (95% CI 0.066 to 0.264, *p* = 0.0018); after adding baseline MMD on the same 39 participants, it was 0.165 (95% CI 0.064 to 0.266, *p* = 0.0021). The absolute change was +0.00061 mm^3^ per point (+0.37%). The focal estimate remained similar in the common *n* = 37 cohort, under six-outcome HC3 inference, after excluding the maximum-Cook observation, and with Huber regression (Table 3; Supplementary Tables S1, S3). The maximum Cook’s distance was 0.119; two observations exceeded the conventional 4/n threshold of 0.103. Excluding the maximum-Cook observation yielded *β* = 0.170 (95% CI 0.070 to 0.269, *p* = 0.0015). Leave-one-out coefficients ranged from 0.150 to 0.178 and remained positive. Residual normality and heteroscedasticity tests did not indicate major violations (Shapiro–Wilk *p* = 0.110; Breusch-Pagan F-test *p* = 0.543), and the maximum variance inflation factor was 1.13. Models adding age squared, HADS, BMI, or menstrual-related headache produced similar coefficients (range, 0.159–0.180; Supplementary Table S3).

**Table 3:** Sequential and robustness analyses for anterior-superior volume.

Analysis	n	β (95% CI), mm^3^ per point	*p*	Notes
Age + eTIV	39	0.165 (0.066 to 0.264)	0.0018	Same 39 participants; sequential model
Age + eTIV + baseline MMD	39	0.165 (0.064 to 0.266)	0.0021	Main model; adjusted *p* = 0.013
Common QC-valid cohort	37	0.156 (0.049 to 0.262)	0.0056	Six-outcome sensitivity; adjusted *p* = 0.033
HC3 inference	39	0.165 (0.082 to 0.249)	<0.001	Six-outcome sensitivity; adjusted *p* = 0.0018
Excluding maximum-Cook observation	38	0.170 (0.070 to 0.269)	0.0015	Selected-outcome influence check
Huber robust regression	39	0.164 (0.062 to 0.266)	0.0016	Selected-outcome robustness check

For linear models, β is the adjusted volume difference in mm^3^ per 1-point higher VSS-C total score. The first two rows use the same 39 participants. The HC3 and common-cohort rows retain six-outcome multiplicity correction; the other rows are selected-outcome robustness checks.

## Discussion

In our reading, the analysis identifies one bounded finding: VSS-C total symptom burden was associated with anterior-superior hypothalamic volume in women with migraine. Among the six software-defined outcomes, only the anterior-superior model met the BH-FDR criterion, and its association persisted when all outcomes were restricted to the same QC-valid 37-participant cohort. This is a subregional statistical association, not evidence of a formally demonstrated difference between subregions or a diagnosis-specific effect: VSS-C total score combines vertigo-balance and autonomic-anxiety symptom frequency and does not establish vestibular migraine or a purely vestibular effect.

Dizziness and vestibular symptoms occur across migraine phenotypes, including outside formal vestibular migraine (1–5). VSS-C and DHI capture related but non-identical constructs: symptom frequency and dizziness-related disability, respectively (19–21). Their convergence on the anterior-superior region is therefore informative, but the strong correlation between the scores and attenuation after mutual adjustment argue against treating DHI as independent replication or assigning separate mechanisms to the two measures.

The anterior-superior location offers an anatomically defined target for follow-up. Within the Billot/FreeSurfer scheme, this label is an atlas-defined anterior hypothalamic compartment and cannot resolve individual nuclei or cellular processes (24, 25). Prior studies have implicated anterior hypothalamic regions in headache disorders, while migraine imaging and theoretical work links the hypothalamus more broadly to homeostatic, autonomic, and interoceptive regulation (6–14, 26–28). These regional functional associations cannot be attributed to a specific nucleus from the present atlas. The result instead narrows a broad hypothalamic hypothesis to a software-defined compartment that can be examined prospectively.

The magnitude and robustness checks help delimit the finding. Adding baseline MMD changed the focal coefficient by only 0.37% on the same denominator, and the estimate remained positive in every leave-one-out refit. Across the observed 20-point VSS-C interquartile range, the model implied a 3.31-mm^3^ difference, equivalent to 8.54% of mean anterior-superior volume. This translation describes the scale of the statistical association; it does not by itself establish clinical importance.

The principal phenotype limitation is that the study analyzed a composite score rather than a formally adjudicated vestibular-migraine cohort or a pure vestibular construct. Item-level and subscale responses were unavailable, preventing separation of vertigo-balance and autonomic-anxiety components. Standardized vestibular testing and participant-level adjudication were also unavailable, so peripheral vestibular and functional dizziness disorders remain competing explanations. Several migraine, diary, treatment, attack-state, and reproductive-hormone fields were unavailable or not sufficiently harmonized for modeling.

All analyzed volumes were derived from a single documented FreeSurfer route, and all 39 retained cases underwent blinded review with independent neuroradiologist confirmation. Two local right anterior-inferior label exceptions were handled prospectively as unavailable for that bilateral outcome rather than repaired or interpreted as biological zeros. Visual QC can exclude grossly unusable segmentations but does not quantify measurement reliability for small subunits. The modest sample also limits precision and makes external replication important.

Finally, the cohort was single-center and restricted to women, so the result cannot be generalized to men or used to infer sex differences. The parent-cohort screening denominator and earlier selection pathway were unavailable, and requiring complete questionnaire, covariate, and usable imaging data may have introduced selection bias. Cross-sectional data cannot distinguish predisposition, consequence, or a shared determinant of VSS-C burden and hypothalamic volume.

Among the six assessed outcomes, the anterior-superior association provides a focused target for prospective replication. A stronger follow-up design would retain continuous symptom measurement while adding item- or component-level VSS-C data, formal vestibular phenotyping, prospectively recorded attack and treatment state, and repeated or independently validated structural measurements.

## Conclusion

In women with migraine, higher VSS-C total symptom burden was associated with larger anterior-superior hypothalamic volume after adjustment for age, eTIV, and baseline MMD and correction across six hypothalamic outcomes. The association remained in the common QC-valid cohort and across the reported robustness checks. These data identify the anterior-superior compartment as a testable target for replication; they do not establish a vestibular-migraine biomarker, nucleus-level mechanism, or causal pathway.

## Data Availability

The original contributions presented in the study are included in the article/Supplementary material, further inquiries can be directed to the corresponding authors.

## Glossary

BH-FDR: Benjamini-Hochberg false-discovery-rate
BMI: body mass index
CI: confidence interval
DHI: Dizziness Handicap Inventory
HADS: Hospital Anxiety and Depression Scale
HC3: heteroscedasticity-consistent type 3
ICHD-3: International Classification of Headache Disorders-third edition
IQR: interquartile range
MIDAS: Migraine Disability Assessment
MMD: monthly migraine days
MRI: magnetic resonance imaging
OLS: ordinary least squares
PSQI: Pittsburgh Sleep Quality Index
QC: quality control
SAGER: Sex and Gender Equity in Research
SD: standard deviation
STROBE: Strengthening the Reporting of Observational Studies in Epidemiology
eTIV: estimated total intracranial volume
VSS: Vertigo Symptom Scale
VSS-C: Chinese version of the Vertigo Symptom Scale
