# Supplementary material for: Hypothalamic subregional volume in relation to VSS-C total symptom burden in women with migraine: an exploratory cross-sectional MRI study
Source: Front Neurol. 2026 Sep 21;17:1931533. doi: 10.3389/fneur.2026.1931533 (PMC13634837; doi:10.3389/fneur.2026.1931533)
Supplement: Supplementary file 1 [file Supplementary_file_1.docx]

Supplementary material

Hypothalamic subregional volume in relation to VSS-C total symptom burden in women with migraine: an exploratory cross-sectional MRI study

This supplement provides supporting analyses, model diagnostics, and an inventory of unavailable or non-harmonized clinical fields. The primary and supporting analyses use hypothalamic volumes generated by the uniform workflow described in the Methods; historical estimates are shown separately for comparison in Table S5.

# Supplementary Table S1. Robustness of the six primary VSS-C models

| **Model/outcome** | **n** | **β (95% CI)** | **P** | **BH-FDR-adjusted P** |
| --- | --- | --- | --- | --- |
| HC3: Anterior-superior | 39 | 0.165 (0.082 to 0.249) | <0.001 | 0.0018 |
| HC3: Anterior-inferior | 37 | 0.018 (-0.149 to 0.186) | 0.824 | 0.907 |
| HC3: Superior tubular | 39 | 0.231 (-0.123 to 0.584) | 0.193 | 0.29 |
| HC3: Inferior tubular | 39 | 0.498 (-0.153 to 1.150) | 0.129 | 0.258 |
| HC3: Posterior | 39 | 0.024 (-0.383 to 0.431) | 0.907 | 0.907 |
| HC3: Whole hypothalamus | 39 | 0.971 (-0.232 to 2.173) | 0.110 | 0.258 |
| Common n=37: Anterior-superior | 37 | 0.156 (0.049 to 0.262) | 0.0056 | 0.033 |
| Common n=37: Anterior-inferior | 37 | 0.018 (-0.108 to 0.145) | 0.768 | 0.922 |
| Common n=37: Superior tubular | 37 | 0.257 (-0.251 to 0.764) | 0.310 | 0.465 |
| Common n=37: Inferior tubular | 37 | 0.512 (-0.158 to 1.182) | 0.130 | 0.336 |
| Common n=37: Posterior | 37 | 0.020 (-0.414 to 0.455) | 0.924 | 0.924 |
| Common n=37: Whole hypothalamus | 37 | 0.963 (-0.427 to 2.354) | 0.168 | 0.336 |

Note. HC3 inference used outcome-specific QC denominators. The common-cohort analysis used the same 37 participants for all six outcomes. BH-FDR correction was applied separately within each six-outcome analysis.

# Supplementary Table S2. Supporting DHI analyses and overlap with VSS-C

| **Model/outcome** | **n** | **β (95% CI)** | **P** | **BH-FDR-adjusted P** |
| --- | --- | --- | --- | --- |
| DHI family: Anterior-superior | 39 | 0.114 (0.042 to 0.185) | 0.0027 | 0.016 |
| DHI family: Anterior-inferior | 37 | -0.005 (-0.092 to 0.082) | 0.908 | 0.908 |
| DHI family: Superior tubular | 39 | 0.214 (-0.118 to 0.546) | 0.200 | 0.299 |
| DHI family: Inferior tubular | 39 | 0.329 (-0.116 to 0.773) | 0.142 | 0.285 |
| DHI family: Posterior | 39 | 0.038 (-0.253 to 0.329) | 0.791 | 0.908 |
| DHI family: Whole hypothalamus | 39 | 0.708 (-0.214 to 1.631) | 0.128 | 0.285 |
| Mutual: VSS-C adjusted for DHI | 39 | 0.104 (-0.026 to 0.234) | 0.113 | — |
| Mutual: DHI adjusted for VSS-C | 39 | 0.067 (-0.025 to 0.158) | 0.147 | — |
| Spearman VSS-C/DHI correlation | 39 | ρ=0.661 | <0.001 | — |

Note. DHI formed a separate supporting six-outcome BH-FDR family. Mutual adjustment and Spearman correlation assess construct overlap and are not independent replication.

# Supplementary Table S3. Diagnostics and selected sensitivity analyses for the anterior-superior model

| **Check or analysis** | **n** | **Result** | **P** |
| --- | --- | --- | --- |
| Maximum Cook's distance | 39 | 0.119; 4/n=0.103; 2 observations above threshold | — |
| Maximum leverage | 39 | 0.369 | — |
| Shapiro-Wilk residual-normality test | 39 | W=0.954 | 0.110 |
| Breusch-Pagan heteroscedasticity F test | 39 | No major heteroscedasticity signal | 0.543 |
| Maximum variance inflation factor | 39 | 1.129 | — |
| Leave-one-out refits | 39 | β range 0.150 to 0.178; all positive | — |
| Exclude maximum-Cook observation | 38 | β=0.170 (0.070 to 0.269) | 0.0015 |
| Huber robust regression | 39 | β=0.164 (0.062 to 0.266) | 0.0016 |
| Primary OLS | 39 | β=0.165 (0.064 to 0.266) | 0.0021 |
| Add age squared | 39 | β=0.166 (0.061 to 0.271) | 0.0028 |
| Add HADS | 39 | β=0.168 (0.067 to 0.269) | 0.0018 |
| Add BMI | 39 | β=0.159 (0.059 to 0.259) | 0.0027 |
| Add menstrual-related headache | 38 | β=0.180 (0.077 to 0.284) | 0.0012 |

Note. The primary model adjusted for age, eTIV, and baseline MMD. Selected-outcome checks assess stability and do not constitute an additional multiplicity-controlled family.

# Supplementary Table S4. Clinical fields unavailable or not harmonized for modeling

| **Field** | **Availability** | **Analytic consequence** |
| --- | --- | --- |
| Formal vestibular-migraine adjudication | Unavailable | VSS-C total is not a diagnostic classification |
| Aura status | Not harmonized | No subtype analysis |
| Episodic/chronic migraine diagnosis | Not harmonized | Baseline MMD reported continuously |
| Medication-overuse headache | Not harmonized | No medication-overuse adjustment |
| Exact peri-ictal phase | Unavailable | Participant-level timing not modeled |
| Acute medication class | Not harmonized | No class-specific adjustment |
| Diary-validity threshold | Unavailable | Baseline MMD treated as recorded |
| Treatment exposure during diary interval | Unavailable | Residual treatment-context confounding possible |
| Current preventive treatment at MRI | Not harmonized | History only; no current-treatment model |
| Menstrual-cycle phase | Unavailable | No phase-specific analysis |
| Hormonal contraception | Unavailable | Not modeled |
| Hormone therapy | Unavailable | Not modeled |
| Menopausal status | Unavailable | Not modeled |

Note. Unavailable fields were not inferred or reconstructed.

# Supplementary Table S5. Comparison of primary VSS-C estimates from historical and uniformly processed volumes

| **Outcome** | **Historical n** | **Historical β (95% CI)** | **Uniform n** | **Uniform β (95% CI)** |
| --- | --- | --- | --- | --- |
| Anterior-superior | 39 | 0.170 (0.070 to 0.270) | 39 | 0.165 (0.064 to 0.266) |
| Anterior-inferior | 39 | 0.101 (-0.028 to 0.230) | 37 | 0.018 (-0.108 to 0.145) |
| Superior tubular | 39 | 0.262 (-0.182 to 0.706) | 39 | 0.231 (-0.248 to 0.709) |
| Inferior tubular | 39 | 0.428 (-0.244 to 1.099) | 39 | 0.498 (-0.133 to 1.130) |
| Posterior | 39 | -0.054 (-0.661 to 0.553) | 39 | 0.024 (-0.392 to 0.439) |
| Whole hypothalamus | 39 | 0.907 (-0.526 to 2.339) | 39 | 0.971 (-0.348 to 2.290) |

Note. β is the volume difference in mm³ per VSS-C total-score point. Both analyses adjusted for age, the same retained eTIV values, and baseline MMD. Historical estimates include the earlier arithmetic reconstruction of four missing components and are shown only for comparison; none of those reconstructed values was used in the current analysis. The uniform-processing estimates are the primary results. For anterior-inferior volume, the comparison includes both regenerated measurements and a denominator change from 39 to 37, so it does not isolate processing effects in a constant sample. This is a descriptive comparison of existing estimates, not an additional hypothesis-testing family. CI, confidence interval; eTIV, estimated total intracranial volume; MMD, monthly migraine days; VSS-C, Chinese version of the Vertigo Symptom Scale.

# Supplementary note. Historical export arithmetic audit

Before uniform reprocessing, arithmetic identities in the retained historical export were checked. The left-side identity held in 39/39 rows and the right-side identity in 35/35 complete rows (maximum absolute residual, 5.68 × 10⁻14 mm³); subtraction returned 0 mm³ for four historical blanks. Because uniform reprocessing yielded positive volumes for three of those four components, none of the reconstructed historical values was used in the current analysis.

**Supplementary Figure S1. Participant flow, uniform processing, and analysis denominators.** The parent-cohort screening denominator and earlier selection pathway were unavailable. The two local label exceptions affected only bilateral anterior-inferior availability; other outcomes remained eligible.

**Supplementary Figure S2. Residual-versus-fitted display for the primary anterior-superior VSS-C model.** The model adjusted for age, eTIV, and baseline MMD (n=39).
